# Post-stroke Hemiplegic Gait: New Perspective and Insights

**DOI:** 10.3389/fphys.2018.01021

**Published:** 2018-08-02

**Authors:** Sheng Li, Gerard E. Francisco, Ping Zhou

**Affiliations:** ^1^Department of Physical Medicine and Rehabilitation, University of Texas Health Science Center, Houston, TX, United States; ^2^TIRR Memorial Hermann Research Center, TIRR Memorial Hermann, Houston, TX, United States; ^3^Guangdong Work Injury Rehabilitation Center, Guangzhou, China

**Keywords:** gait, stroke, hemiparesis, spasticity, botulinum toxin, motor recovery

## Abstract

Walking dysfunction occurs at a very high prevalence in stroke survivors. Human walking is a phenomenon often taken for granted, but it is mediated by complicated neural control mechanisms. The automatic process includes the brainstem descending pathways (RST and VST) and the intraspinal locomotor network. It is known that leg muscles are organized into modules to serve subtasks for body support, posture and locomotion. Major kinematic mechanisms are recognized to minimize the center of gravity (COG) displacement. Stroke leads to damage to motor cortices and their descending corticospinal tracts and subsequent muscle weakness. On the other hand, brainstem descending pathways and the intraspinal motor network are disinhibited and become hyperexcitable. Recent advances suggest that they mediate post-stroke spasticity and diffuse spastic synergistic activation. As a result of such changes, existing modules are simplified and merged, thus leading to poor body support and walking performance. The wide range and hierarchy of post-stroke hemiplegic gait impairments is a reflection of mechanical consequences of muscle weakness, spasticity, abnormal synergistic activation and their interactions. Given the role of brainstem descending pathways in body support and locomotion and post-stroke spasticity, a new perspective of understanding post-stroke hemiplegic gait is proposed. Its clinical implications for management of hemiplegic gait are discussed. Two cases are presented as clinical application examples.

## Introduction

Stroke is a leading cause of serious long-term disability (Benjamin et al., 2017). Walking dysfunction occurs in more than 80% of stroke survivors (Duncan et al., 2005). Despite of rehabilitation efforts, 25% of all stroke survivors have residual gait impairments that require full physical assistance before hospital discharge (Hendricks et al., 2002). Consequently, gait impairments cause difficulties in performing activities of daily living and mobility. Gait abnormality is characterized by a pronounced clinical presentation of gait asymmetry, as compared to healthy people (Olney and Richards, 1996; Richards and Olney, 1996). Stroke survivors usually have decreased stance phase and prolonged swing phase of the paretic side. Further, the walking speed is decreased and the stride length is shorter (Perry and Burnfield, 2010). These gait abnormalities along with muscle weakness place stroke survivors at a high risk of falls (Dobkin, 2005; Batchelor et al., 2012). Falls usually occur during walking in community-dwelling stroke survivors (Hyndman et al., 2002). Thus, improving walking safety and speed is the major goal for stroke survivors to prevent falls and to improve quality of life (Olney and Richards, 1996; Dobkin, 2005).

Walking is a phenomenon that is taken for granted by healthy individuals but requires an extremely complex process of neuromusculoskeletal control. Activation of muscles in lower limbs, trunk, and upper limbs in a certain spatiotemporal pattern is required to ensure appropriate joint positions to support and advance the body weight in different phases of gait cycles. In most situations, human walking at a comfortable speed on the level surface is primarily mediated by brainstem and spinal mechanisms (Dietz, 1996; Nielsen, 2003). However, supraspinal control adds complexity and flexibility of gait control and gait versatility to meet dynamic environmental needs and challenges (Dietz, 1996; Nielsen, 2003). Spasticity and paresis are main motor impairments after stroke (Li, 2017). In the context of spastic hemiparesis, muscles are weak and spastic and at different levels of impairments involving different regions of the upper limb, trunk and lower limb on one side. As a result, a wide spectrum of gait abnormalities is seen clinically.

In this article, major kinematic determinants and neural control of normal human gait are briefly reviewed from a historical perspective. Current findings of post-stroke hemiplegic gait as a result of altered neural control are then summarized. Based on recent advances on pathophysiology of muscle weakness and spasticity after stroke, a new perspective of understanding post-stroke hemiplegic gait is proposed. Its clinical implications for management of hemiplegic gait are discussed.

## Major kinematic determinants of normal human gait

For a biomechanical and kinesiological point of view, human walking can be described as progression of alternating weight-bearing limbs. As such, the displacement of the center of gravity (COG) of the whole-body is viewed as the end result of all muscle forces acting upon the body during the progression. During normal level walking, the body COG follows a smooth regular curve in the three-dimensional space. The peak-to-peak amplitudes are ~5 cm in the vertical and mediolateral planes, respectively Saunders et al. (1953). Using a hypothetical bipedal compass gait model and elementary geometrical arguments, Saunders et al. (1953) proposed six kinematic mechanisms that contribute to the efficient progress of the whole-body COG in the three dimensional space. These mechanisms are termed as six major determinants of human gait. They include pelvic rotation in the transverse plane, pelvic tilt in the coronal plan, knee flexion in the stance phase, foot and knee mechanisms and lateral displacement of the pelvis (hip adduction). This concept of major determinants was originally proposed to understand and manage pathological gait after orthopedic disorders, such as a fused hip joint (Saunders et al., 1953). From a historical perspective, major determinants of human gait are the fundamental concepts in understanding control of human gait and providing a foundation for clinical application of gait analysis. Although individual muscle activities (electromyography, EMG), joint kinematics, and ground reaction force were not available in the original “compass gait” model that permits only hip flexion and extension during walking, these determinants were able to explain the minimization of COG displacement well.

The conclusion of six determinants of human gait has been challenged in a number of studies (Gard and Childress, 1997, 1999; Croce et al., 2001; Kuo, 2007; Hayot et al., 2013). In the most recent study (Lin et al., 2014), Lin et al. quantitatively assessed the contribution of each determinant to the COG displacement over a gait cycle in young and healthy people. Using an “influence coefficient” concept, they found that hip flexion, stance knee flexion, and ankle-foot interaction significantly minimized the COG displacement in the sagittal plane; hip adduction and pelvic tilt are the main determinants of the mediolateral COG displacement in the coronal plane; however, pelvic rotation and pelvic tilt do not significantly affect the vertical COG displacement. Overall, there is general agreement between Saunders et al.'s classic article and this study with comprehensive quantitative kinematic data of individual joints. It is confirmatory that pelvic girdle movements (pelvic tilt, hip flexion, and adduction) contribute significantly to the displacement of COG in the three-dimensional space during walking.

## Neural control of normal human gait

The above kinematic mechanisms are not able to account for a near perfect kinematic trajectory during human walking on a level surface, however. The distal part of the foot in the swing phase is lifted only 1–2 cm with <4 mm step-to-step variations (Winter, 1992). This displacement is enough to prevent stumbling, but not more than necessary. This remarkable precision of the foot position in the swing phase is determined by and the end result of coordinated activation of muscles from the lower extremities directly and of trunk and arm muscles indirectly. The number of different combinations of muscle activations that lead to the same foot position is almost infinite, i.e., the problem of motor redundancy (Bernstein, 1967). As suggested by Bernstein (1967), the brain may only control the endpoint, i.e., the foot position in this case, while allowing considerable flexibility for specific muscle activities. Using this fundamental approach, the muscle activities are not controlled individually. They are allowed to have a large range of flexibility as long as they are all scaled to each other to ensure the endpoint: the foot position within a desired range. These muscles are coordinated and organized into functional groups. They are often referred as muscle synergies or modules (Ting and McKay, 2007; Drew et al., 2008).

Different modules are described according to their biomechanical functions to the whole limb or the whole body during different types of locomotor functions, such as balance control or walking (Beyaert et al., 2015). There are five modules that are sufficient to perform sub-tasks of walking (Neptune et al., 2009). Module 1 includes gluteus medius, vasti, and rectus femoris muscles, primarily contributing to body support in early stance. Module 2 (soleus and gastrocnemius) is activated during both body support and propulsion in late stance. Module 3 (rectus femoris and tibialis anterior) acts to decelerate the leg in early and late swing, as well as to generate energy to the trunk throughout the swing phase. Module 4 mainly consists of the hamstring muscles. Activation of these muscles decelerates the ipsilateral leg prior to heel strike. Module 3 and Module 5 (iliopsoas) act together to accelerate the ipsilateral leg forward in early swing. These modules represent a general repertoire of motor actions that can be recruited in a variety of combinations and at different times for different locomotion and balance control needs, as well as for voluntary, rhythmic and reactive locomotor behaviors (McGowan et al., 2010; Allen and Neptune, 2012; Beyaert et al., 2015).

Extensive neural structures and pathways are involved in the process of gait control, including the spinal cord, brainstem, cerebellum, basal ganglia, limbic system, and cerebral cortex, as well as their interactions with the environment (see review Nielsen, 2003; Beyaert et al., 2015). Briefly, the above motor modules are largely controlled by the spinal cord and brainstem under regulating control of the cerebellum. More specifically, the pontine medullary reticular formation (PMRF) and vestibular nuclei provide body support and balance control, thus providing an upright posture against gravity by activating trunk and lower extremity extensor muscles. The additional neurons in the PMRF activate the spinal locomotor network under influence of the mesencephalic locomotor region and subthalamic locomotor region or cerebellum. Activation of this network allows rhythmic locomotor activity. These structures constitute automatic processes by simultaneously controlling body support, balance and rhythmic locomotor activity. However, locomotion occurs only when this automatic process is initiated “volitionally” or “emotionally.” The volitional process involves the cerebral cortex while an emotional process involves the limbic system. The basal ganglia influence volitional, emotional and automatic processes through its interactions with the cerebral cortex, limbic system, and brainstem, respectively. Furthermore, real-time sensory feedback via visual signals, vestibular, and proprioceptive signals is crucial for locomotor adaptation. In summary, walking is mainly a result of automatic process, involving the spinal cord and brainstem mechanisms. It is usually achieved and maintained without conscious awareness and cognitive processing.

## Altered neural control and pathomechanics of post-stroke hemiplegic gait

Neural control mechanisms are altered in stroke survivors with walking dysfunction. As compared to normal healthy controls, stroke survivors have fewer modules during walking (Clark et al., 2010). In their study (Clark et al., 2010), Clark and colleagues analyzed modules based on EMG signals from eight leg muscles in 55 subjects with chronic stroke and in 20 controls. Most of affected legs had only just two or three modules. These modules were merged from the modules observed in control subjects, thus less independent neural control for affected leg. Furthermore, the authors reported that the number of simplified modules was correlated to preferred walking speed, speed modulation, step length asymmetry, and propulsive asymmetry. In other words, stroke survivors with fewer modules on the paretic limb walk more slowly and demonstrate more gait asymmetry (Routson et al., 2014). This modification of modular organization likely reflects the central nervous system's response to muscle weakness and lack of voluntary muscle control on the affected side to improve body support and locomotion. In addition to simplified modular organization, abnormal muscle synergies and spastic synergistic activation patterns are often resulted as well (Kline et al., 2007; Finley et al., 2008). For example, Finley et al. demonstrated a reflex-mediated coupling between hip flexion and knee extension in stroke survivors (Finley et al., 2008). As a result of abnormal patterns of muscle activation, joint positions are altered at rest and joint movements are coupled during walking.

A full spectrum of gait abnormality is observed clinically, depending on the level of muscle weakness, severity of spasticity, compensatory mechanisms, and their interactions. Primarily due to muscle strength on the paretic side, there is a hierarchy of gait impairments. According to walking speeds which correspond to muscle weakness, stroke survivors are classified into four groups with different features of gait impairments (Mulroy et al., 2003). They are: Fast walker, Moderate walker, Slow-Extended walker (circumductory gait), and Slow-Flexed walker.

In the Fast walker group, a stroke survivor has ~44% of a normal walking speed. There is a lack of heel rise in the terminal stance, due to inadequate plantarflexor (PF) muscle strength. Otherwise, discriminating gait events are within normal limits. Knee hyperextension in the stance phase is observed to compensate for lack of heel rise so that the body can roll forward onto the forefoot. As such, the step length is compromise secondary to lack of transition of momentum from the unaffected limb.

A typical Moderate walker has ~21% of a normal walking speed. The stroke survivor is able to walk without any assistance. The plantar flexor muscles on the paretic side are further weakened. There are some weakness in hip extensors (gluteus maximum) and knee extensors (quadriceps muscle). Along with weakness, Gluteus maximum muscles, quadriceps, and plantarflexors start to show spastic responses to quick stretch. As a result, excessive knee flexion and hip flexion occur at the mid stance phase. Due to the lack of pre-swing forward progression over the toe rocker, ankle plantar flexion, knee flexion, and heel-off are inadequate in the terminal stance. However, the survivor is still able to achieve a neutral foot position for clearance in the mid swing phase.

In the Slow-Extended walker group, quadriceps muscles are further weakened, and are not able to support the knee during the stance phase. Though weak, the gluteus maximus muscle is still strong enough to retract the femur into knee hyperextension to support the body. There are also some plantarflexors contracture and spasticity to provide necessary ankle stability. During the swing phase, there is persistent gluteus maximum and ankle plantarflexor spasticity. Hip hiking and leg circumduction occur for foot clearance. Stroke survivors in this group usually require assistive devices to walk. The walking speed is further decreased at ~11% of a normal speed.

In the Slow-Flexed walker group, the gluteus maximus muscle is weakened further to the extent that it is not able to retract the femur to stabilize the knee. Strength limitation across hip, knee and ankle joints leaves stroke survivors with the boardline walking ability. In the mid stance, there is excessive hip and knee flexion, ankle dorsiflexion, and trunk forward leaning. This posture persists in the swing phase with assistance. The assisted walking speed is at about 10% of a normal speed.

## Pathophysiology of hemiparesis and spasticity after stroke

Spasticity and muscle weakness (i.e., spastic paresis) are the primary motor impairments and impose significant challenges for patient care. Spasticity is estimated to be present in about 20–40% of stroke survivors (Zorowitz et al., 2013). Clinically, post-stroke spasticity is easily recognized as a phenomenon of velocity-dependent increase in tonic stretch reflexes (“muscle tone”) with exaggerated tendon jerks, resulting from hyperexcitability of the stretch reflex (Lance, 1980). Based on decades of animal studies and recent human research (Brown, 1994; Gracies, 2005; Nielsen et al., 2007; Mukherjee and Chakravarty, 2010; Burke et al., 2013; Stecco et al., 2014; Li and Francisco, 2015), there are advances in understanding the pathophysiology of spasticity and its relation with paresis (Li and Francisco, 2015; Li, 2017). A brief summary is presented here. In a stroke survivor with spastic hemiplegia, damages occur to the motor cortices and their descending corticospinal tract (CST). These damages cause muscle weakness (usually hemiparesis) immediately after stroke, including upper extremity, trunk, and lower extremity muscles on the affected side. On the other hand, neuroplasticity occurs after stroke as well. Due to lesions of corticobulbar pathways accompanied with lesion of motor cortices and/or descending CST, bulbospinal hyperexcitability develops due to loss of supraspinal inhibition. This is mainly a phenomenon of disinhibition, or unmasking effects. There are several potential candidates, including reticulospinal (RST), vestibulospinal (VST), and rubrospinal projections (Miller et al., 2014; Li and Francisco, 2015; Owen et al., 2017). Medial RST hyperexcitability appears to be the most likely mechanism related to post-stroke spasticity (Li and Francisco, 2015). RST hyperexcitability provides unopposed excitatory descending inputs to spinal stretch reflex circuits, resulting in elevated excitability of spinal motor neurons. This adaptive change can account for most clinical findings on spasticity, for example, exaggerated stretch reflex, velocity-dependent resistance to stretch, muscle overactivity, or spontaneous firings of motor units. Spasticity usually leads to a synergistic pattern of activation during standing and walking, e.g., flexor synergy in the upper extremity and extensor synergy in the lower limb (Francisco and Li, 2016). The inter-limb activation coupling between upper and lower extremities is also reported (Kline et al., 2007).

## A new perspective for understanding hemiplegic gait

**Figure 1:**
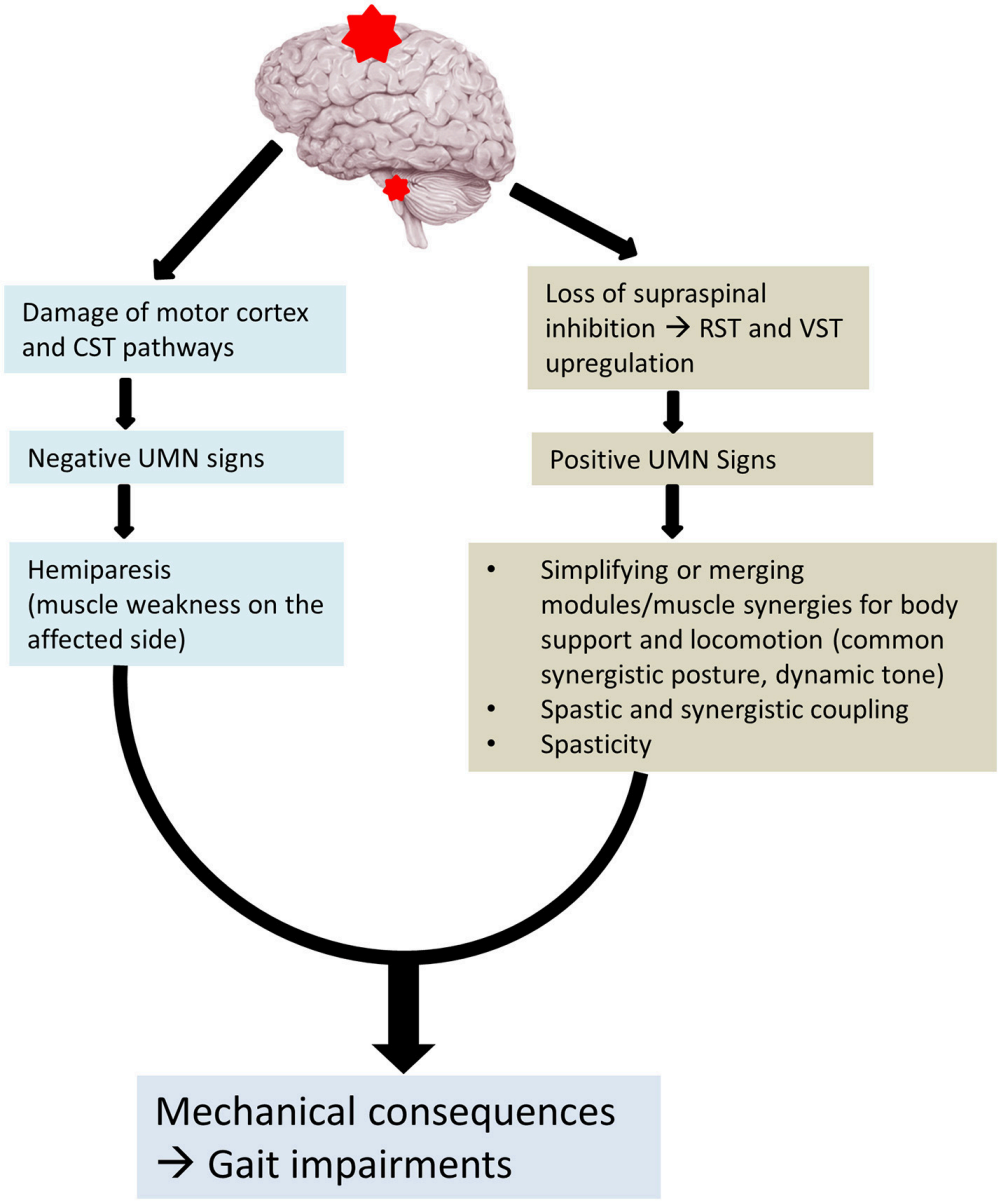
Altered neural control for post-stroke gait. CST, corticospinal tract; RST, reticulospinal tract; VST, vestibulospinal tract.

These recent advances in understanding the pathophysiology of spasticity and its relations to muscle weakness can help us better understand hemiplegic gait in stroke survivors. Given the disinhibited brainstem descending pathways (RST and VST) are linked to post-stroke spasticity, reorganization of modular control, and spastic synergistic activation, a new perspective for understanding hemiplegic gait is schematically illustrated in Figure 1. Muscle weakness is primarily a result of damage to motor cortices and their descending CST after stroke. Muscle strength, especially knee extensor strength determines gait independence (Akazawa et al., 2017). Disinhibited brainstem descending pathways (RST and VST) are hyperexcitable. These descending projections are diffuse and the activated muscles are organized into fewer modules or motor synergies that provide body support and posture stability and locomotion (Nielsen, 2003; Beyaert et al., 2015). In addition, they also mediate spasticity and spastic synergistic patterns. The most commonly observed abnormal patterns include flexor synergies in the upper extremity and extensor synergies in the lower extremity. These spastic activations also lead to abnormal coupling within a limb (Finley et al., 2008) and between limbs (Kline et al., 2007). The interactions among muscle weakness, spasticity, and spastic activations act on the trunk, pelvis and the legs. Mechanical consequences of these interactions are the clinically observed gait impairments. They are exemplified in the stereotypical hemiplegic gait. It is usually described as hip extension, adduction, and medial rotation, knee extension, ankle plantar flexion, and inversion. The spastic muscles are synergistically activated into hip and knee extension during the stance phase of walking. The abnormal activation does not allow the hip and knee to flex for foot clearance. To compensate for these impairments, stroke survivors usually hike hip and circumduct the affected leg during the swing phase for foot clearance. As such it is known as a “circumductory gait.” Depending on the severity of weakness and spasticity, and the degree of involvement (focal, regional, or extensive), a wide spectrum of gait impairments are clinically observed, as described above.

## Implications for management of hemiplegic gait

Improving walking safety and speed is the major goal for gait rehabilitation for stroke survivors to prevent falls and subsequently to improve quality of life (Olney and Richards, 1996; Dobkin, 2005). A multi-modality interdisciplinary approach is usually employed and encouraged to bring the maximum clinical outcomes for stroke survivors. Gait rehabilitation programs include muscle strength training, task-specific gait training, treadmill training, electromechanical and robot-assisted gait training, functional electrical stimulations, ankle foot orthoses (AFOs), virtual reality, mental practice with motor imagery, and botulinum toxin injection of spastic muscles (Verma et al., 2012; Tenniglo et al., 2014; Beyaert et al., 2015; Hsu et al., 2017; Jacinto and Reis Silva, 2018). The proposed new perspective also has clinical implications to improve management of hemiplegic gait. A few areas are discussed here as examples.

### Spastic kinetic chain and orthotic management

As outlined above and in Figure 1, gait abnormality is a mechanical consequence of altered neural control after stroke. Abnormal joint posture during the stance phase represents the net result of interactions between ground reaction force and activation of spastic paretic muscles. For example, inadequate quadriceps support often results in a unique joint abnormality during the stance phase, i.e., greater knee flexion in the Moderate walker group. This knee position places the ground reaction force further anterior to the ankle joint, posterior to the knee joint, and anterior to the hip joint. In response to the increased moment imposed to each joint of the kinetic chain, spastic activation of gluteus muscles to assist hip extension, of quadriceps muscles to assist knee extension, and of ankle plantarflexors and invertors to assist ankle dorsiflexion and stabilization. For such a spastic kinetic chain, bracing with ankle-foot-orthosis to decrease ankle dorsiflexion angle is likely effective in changing the vector of ground reaction force (Owen, 2010). The forces required for maintaining joint position at each joint are reduced, and body support and joint stability are improved.

### Muscle selection for botulinum toxin therapy

Botulinum toxin therapy is often used for spasticity management of leg muscles to improve gait (Esquenazi et al., 2015; Baker et al., 2016). Botulinum toxin (BoNT) acts to block presynaptic release of acetylcholine at the neuromuscular junction, therefore, intramuscular injection of BoNT can lead to spasticity reduction (Jahn, 2006). Due to this, BoNT injection also results in muscle weakness. As stated above, increased spasticity of quadriceps is likely to be part of synergistic activation for body support and posture stabilization. Quadriceps strength and support determines walking independence (Akazawa et al., 2017). Though quadriceps spasticity is often linked to knee joint stiffness, judicious consideration of treatment for spasticity is required because of the side effect of muscle weakness from BoNT. Another common observation is that stroke survivors have ankle plantarflexion and ankle inversion. Intramuscular EMG exams may detect spontaneous motor unit activation potentials (MUAPs) in most relevant muscles, such as tibialis posterior, gastrocnemius, soleus, tibialis anterior, extensor hallux longus muscles, i.e., spasticity (Mottram et al., 2009, 2010; Chang et al., 2013). It is not surprising to detect spasticity in all of these muscles, given diffused activation of brainstem descending pathways. The clinical presentation of ankle plantarflexion and ankle inversion suggests that this abnormality is primarily caused by tibialis posterior, gastrocnemius, and soleus, or spasticity of these muscles overrides spasticity of tibialis anterior and extensor halluces longus muscles. Not all muscles with spasticity need botulinum toxin injection in this case. Rather, selection of muscles is based on mechanical consequences of spastic muscles and their relation to ankle and foot positioning during walking.

### Muscles for pelvis and posture control

**Figure 2:**
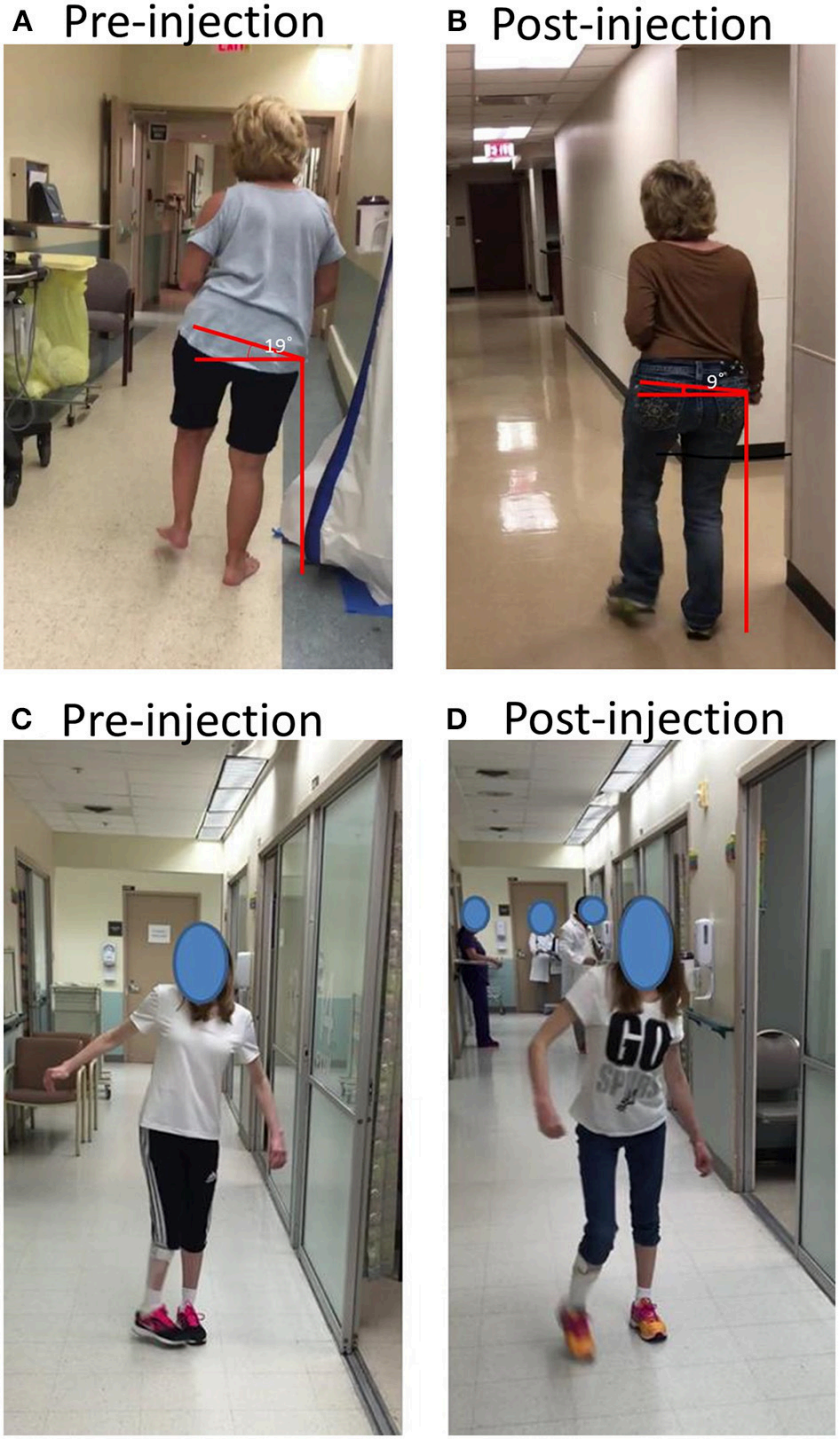
**(A,B)** A stroke survivor with spasticity that resulted in dramatic trunk lateral flexion and hip hiking before and after botulinum toxin injections; **(C,D)** A stroke survivor with spasticity that resulted in dynamic hip adduction and pelvic anterior rotation before and after botulinum toxin injection. See text for details.

Major kinematic determinants were originally proposed to explain contributions of individual joints (pelvic movement, hip, knee, and ankle joints) to minimize the COG displacement. The purpose was to understand human gait in general and to explain gait abnormality after orthopedic disorders in particular, such as hip joint fusion. As mentioned above, these kinematic determinants were in general validated by the modern instrumented gait analysis. Even though three out of six kinematic determinants involve pelvic movement, EMG studies are almost limited to leg muscles. Only one muscle (gluteus maximus) related to pelvic movement is commonly studied (Perry and Burnfield, 2010). The neural control mechanisms (brainstem-spinal network) involve trunk muscles and other pelvic movement related muscles as well. Post-stroke spastic hemiparesis could involve all muscles on the affected side. Depending on clinical presentations, these pelvic muscles could be the primary contributors of the gait impairments (Figure 2). Two cases are presented here to highlight the importance of spastic latissimus dorsi muscle and gluteus medius and tensor fasciae latae (TFL) muscles in post-stroke gait control. Written informed consent was obtained for scientific publication from both patients.

### Case 1

A 62 year old right-handed female suffered right middle cerebral artery ischemic stroke 6 years ago with a residual left spastic hemiplegia. She was able to ambulate without any assistive device at a moderate walking speed. She presented with a mild circumductory gait. Lateral trunk flexion to the left side and her left hip hiking were prominent and constant during walking. According to its spread origin of latissimus dorsi muscle from inferior 3–4 ribs, low thoracic spine, lumbar spine and iliac crest, and its insertion to the intertubercular groove of the humerus, a spastic latissimus dorsi muscle was viewed to be responsible for this patient's abnormal posture during walking, including pelvic vertical elevation in the coronal plane, trunk lateral flexion, shoulder adduction, and internal rotation (Figure 2A). A total of 150 units of onabotulinumtoxin A were injected into this muscle under ultrasound imaging guidance. Trunk lateral flexion and pelvic elevation were much improved at 6 weeks after injection. As shown on Figure 2B, pelvic vertical elevation was decreased from 19 to 9° after injection.

### Case 2

A 27 year old right handed female had a history of stroke after a traumatic brain injury 20 years ago which resulted in right spastic hemiplegia. She received botulinum toxin injections several times in the first 3 years after the accident. At a seated or supine position, she only had very mild muscle weakness in the right upper and lower extremities with minimum to negligible spasticity. The chief complaint was that her right toes were hitting the left toes during the mid-swing phase, i.e., problematic right hip internal rotation and adduction secondary to dynamic tone (Figure 2C). According to possible pathomechanics, dynamic spasticity in right anterior gluteus medius and TFL muscles could cause excessive anterior rotation of the pelvis in the transverse plane and hip internal rotation, while hip adductor spasticity contributes further to hip adduction. A total of 200 units of incobotulinumtoxin A were injected to these muscles under ultrasound imaging guidance (75 units to gluteus medius, 50 units to TFL, and 75 units to hip adductors). Improved walking posture in the follow up visit at 6 weeks after injection validated the pathomechanics analysis (Figure 2D).

## Concluding remarks

Given the disinhibited brainstem descending pathways (RST and VST) are linked to post-stroke spasticity, reorganization of modular control, and spastic synergistic activation, a new perspective for understanding hemiplegic gait is proposed. This new perspective highlights post-stroke hemiplegic gait impairments as mechanical consequences of altered neural control mechanisms of human gait. Hemiplegic gait is not a result of isolated skeletal muscular disorder, as often seen after orthopedic disorders. In clinical observational analysis, muscle weakness, spasticity, and spastic activation on the paretic arm, trunk and leg need to be taken into consideration. This new perspective also advances clinical management strategies as outlined above. However, these are examples and cases. They need to be validated in future laboratory and clinical studies.

## Author contributions

SL developed the initial version of the manuscript and created the figures. GF and PZ critically revised the manuscript and contributed substantially to the manuscript development. All authors read and approved the final manuscript.

### Conflict of interest statement

The authors declare that the research was conducted in the absence of any commercial or financial relationships that could be construed as a potential conflict of interest.
